# Unilateral thalamic infarction onset with lethargy: A case report and literature review

**DOI:** 10.1097/MD.0000000000032158

**Published:** 2022-12-02

**Authors:** Wei Kong, Lei Ma, Changyou Yin, Wei Zhao, Yanbin Wang

**Affiliations:** a Department of Neurosurgery, The Affiliated Yantai Yuhuangding Hospital of Qingdao University, Yantai, China; b Department of Anaesthesiology, The Affiliated Yantai Yuhuangding Hospital of Qingdao University, Yantai, China.

**Keywords:** case report, cerebral infarction, lethargy, reticular nucleus of thalamus, review

## Abstract

Infarct-induced lethargy is a common disabling symptom that lacks a consensual definition and a standardized method of care. Identifying the causes of the infarct in the thalamic reticular nucleus (TRN) induced lethargy is crucial in stroke patients.

**Case presentation:**

A 68-year-old female patient was admitted to the hospital with lethargy and weakness in the right limb. A computed tomography (CT) scan performed at the presentation showed no bleeding. She was given intravenous thrombolysis. A head computed tomography (CT) scan clearly showed that the infarct was located in the TRN. After 1 hour of treatment, the weakness in the patient’s limb was relieved. However, she was still lethargic, but her lethargy symptoms improved after 3 days.

**Discussion and conclusions:**

Our case highlights that despite the small size of the infarct, the patient was unconscious, which makes it difficult for physicians to understand and treat the condition, resulting in trouble managing the case. We performed a literature review and proposed that the infarction located in the TRN causes lethargy. However, further clinical and pathophysiological research is still needed to improve patient care.

## 1. Introduction

Ischemic stroke often occurs in the elderly (50–60 years of age) when clots block cerebral circulation. Major modifiable risk factors for stroke include diabetes mellitus, anemia, hypertension, tobacco use, and dyslipidemia. Some patients experience clinical symptoms of transient ischemic attacks, such as dizziness, temporary limb numbness, and weakness. Cerebral infarction is difficult to diagnose if the infarct site is located in the thalamic reticular nucleus (TRN) region, which induces lethargy in stroke patients, causing a delay in treatment. An emergency diffusion-weighted magnetic resonance imaging (DW-MRI) can be performed to get up early, make a precise diagnosis, and avoid time delays in treatments such as toxicity and metabolism. A bridging mechanical thrombectomy can be performed to improve patient care and prognosis.

## 2. Case presentation

This study was approved by the Institutional Review Board of our institute. A 68-year-old female patient was hospitalized for 4 hours due to lethargy and weakness in the right limb. The patient had a history of diabetes, which was maintained through diet and exercise. She had no previous history of atrial fibrillation. The patient had a temperature of 36.8°C, a pulse of 54 beats/min, a breathing frequency of 15 breaths/min, blood pressure of 151/71 mm Hg, and blood glucose of 7.9 mmol/L at the time of admission.

**Figure 1.**
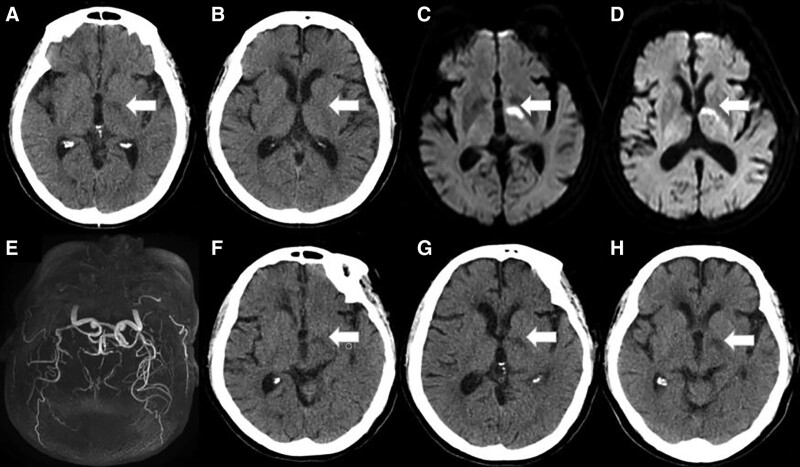
A and B, there is no hemorrhage. A low-density image of the anterior reticular nucleus of the left thalamus can be seen at the level of the third ventricle. C, D shows the limited diffusion of the left thalamic reticular nucleus. E there is no stenosis or occlusion of large blood vessels in the anterior intracranial circulation. F and G images show low-density visualization of the left thalamic reticular nucleus area after thrombolysis, which is slightly larger than before. Only a few low-density areas are left in one CT slice in the H image. CT = computed tomography.

The physical examination results were as follows: lethargy, eyes open when calling, pupils on both sides of the same circle, sensitive light reflection, right lower limb muscle strength level 4 with positive Babinski signs, and normal left limb muscle strength with negative Babinski sign. The sensation of both her limbs and face was symmetrical, and there was no sensory abnormality. A computed tomography (CT) scan performed at the emergency department (Fig. 1A and B) showed no bleeding with a NINSS score of 3. The TOAST classification criteria were small arteriolar occlusion. Intravenous thrombolysis was administered immediately. A 100 U of urokinase was added to 100 mL of 0.9% sodium chloride solution, and the infusion was completed in half an hour. Diffusion-weighted imaging (Fig. 1C and D) was performed after thrombolysis, indicating a limited diffusion of the left TRN area. Electroencephalogram (EEG) was performed without abnormality. Magnetic resonance angiography (Fig. 1E) showed no occlusion of large blood vessels that supply the brain’s anterior circulation. After thrombolysis, CT (Fig. 1F and G) showed a developed left TRN with low density and a slightly larger area than before. Besides, the symptoms of weakness in the right lower limb were improved, but the patient was still in a state of lethargy. She was administered 3-n-butylphthalide (dl-NBP) and edaravone to treat ischemic stroke. The drowsiness symptoms improved after 3 days, the CT results (Fig. 1H) were reexamined after a week, and only a few low-density areas were found on 1 level. The patient’s condition improved, and he was discharged.

## 3. Discussion and conclusions

**Figure 2.**
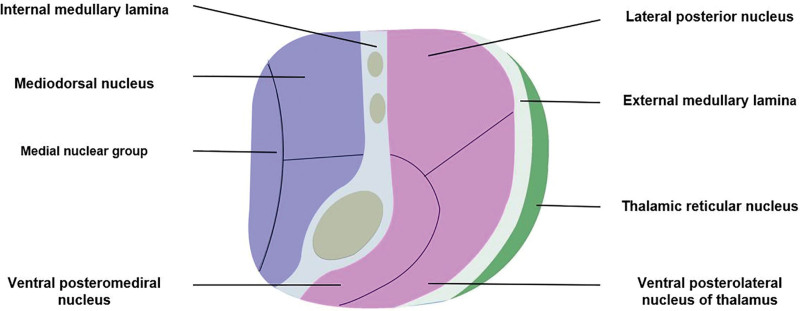
The relationship between the reticular nucleus and the thalamus.

**Figure 3.**
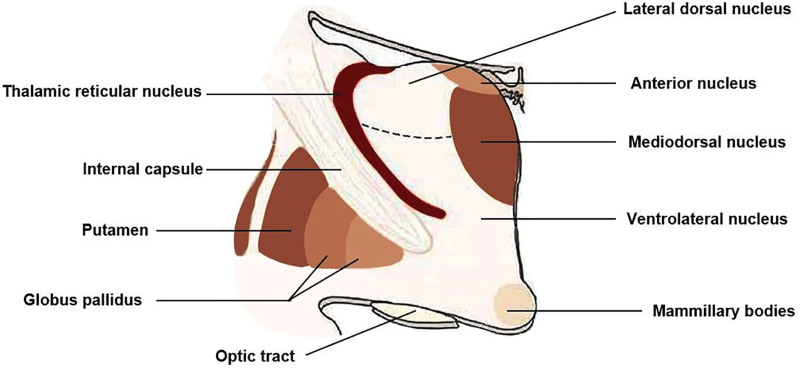
The relationship between the reticular nucleus and surrounding tissues.

The TRN is located next to the white matter of the thalamus, between the inner capsule and the outer medullary plate, surrounding the dorsal and anterior parts of the thalamus (Figs. 2 and 3). TRN is a reservoir of inhibitory GABAergic cells, receives inputs from the thalamic nuclei and cortical and subcortical areas, and emits inhibitory projections to the thalamocortical neurons.^[1,2]^ GABAergic neurons account for 90% of all the neuronal fiber projections of the TRN. Therefore, the TRN plays essential roles in information transmission between the thalamus and the cortex, such as gating,^[3]^ regulating attention,^[4]^ sleep,^[5]^ and sensory processing.^[6]^ Although TRN is often addressed as a homogenous group of GABAergic neurons, recent studies on laboratory animals suggest the existence of TRN neuron heterogeneity, including molecular and electrophysiological properties, connectivity, and function. The 2 TRN subpopulations make a differential connection with the functionally distinct thalamic nuclei to form molecularly defined TRN-thalamus subnetworks. Selective perturbation of the 2 subnetworks in vivo revealed their differential role in regulating sleep. By the targeted ectopic expression of the γ6 regulatory subunit in these TRN subpopulations, we demonstrated their distinct effects on sleep delta and spindle rhythms in vivo.^[7]^ Do not be confused by the name of the reticular nucleus. It is not a continuation of the mesencephalic reticular formation. The TRN accepts cholinergic, monoaminergic, and GABAergic projections from the basal forebrain and brainstem.^[8]^

In clinical practice, we have observed that the destruction of TRN causes hypersomnolence. Studies have found that stimulating selective brainstem projections of cholinergic nerve fibers to TRN can promote the occurrence of sleep.^[3]^ In this case, the patient’s drowsiness was accompanied by weakness of the right lower limb. Cerebral edema is a common complication after ischemic stroke. After recanalization, the weakness was controlled and improved using drugs. However, the core infarct area was located in the TRN, so the patient’s drowsiness symptoms did not improve immediately.

Previous studies have shown that there are no synaptic connections between interneurons in the reticular nucleus of the thalamus.^[9–11]^ The reticular nuclei encapsulate and dominate most of the thalamic nuclei. The glutamatergic neurons of the cortex communicate with other thalamic nuclei through the toilet paper fibers, and the glutamatergic projections of the thalamic-cortical circuit innervate the thalamic reticular nuclei through the collateral fibers. Unlike the other nuclei of the thalamus, the TRN does not project directly to the cerebral cortex through nerve fibers but instead projects dense GABAergic nerve fibers to other brain regions of the thalamus.^[12]^

The low threshold Ca^2 + ^current induced by the periodic phasic discharge of the γ-aminobutyric acid (GABA) neurons in the TRN causes the sleep spindle, representing its starting point.^[13]^ Sleep spindles are thalamocortical oscillations electroencephalogram (EEG) hallmark of the non-rapid eye movement sleep (NREMS). Spindle waves can reduce the impact of external stimulation on the brain during sleep^[14]^ and sleep-dependent memory.^[15]^ Therefore, studies have shown that stimulating the TRN can prolong the time of non-rapid eye movement sleep (NREMS).^[16,17]^

There are 2 main types of cholinergic neurons in the central nervous system. The first type is local loop neurons, which are mainly located in the ventral striatum and the nucleus accumbens nucleus, and are related to the regulation of movement. The second type is projection neurons, mainly distributed in the basal forebrain and brainstem. Cholinergic neurons account for about 5% of all basal forebrain neurons.^[18]^ However, they can widely project to the cortex, hippocampus, and other regions and are related to learning and memory.^[19]^ The cholinergic neurons can project to the thalamus, neostriatum, basal forebrain, and other regions in the brainstem. Their fibers, along with other ascending fibers, form an ascending reticular activation system, which can regulate breathing, biological rhythms, being awake and fast-moving, and maintain the excitement of the cortex during sleep.^[20–25]^ These studies believe that cholinergic projections in the brainstem can facilitate the thalamus sensory area through N-type receptors and inhibit the TRN through M-type receptors. When the GABA neurons in the TRN are activated, they can play the same role in activating the cholinergic nerve endings, promoting sleep initiation, and changing the sleep structure.^[26]^ When the GABA neurons in the TRN are inhibited, the production of sleep spindle waves is inhibited, and the inhibitory effect of the TRN on the thalamus is also inhibited. Then, the thalamus facilitates the transmission of information, such that the cholinergic system plays a positive role in the sleep system.^[27,28]^

Some studies have shown that the occurrence of a cerebral infarction leads to a burst-type neurotransmitter discharge of neurotransmitters.^[29]^ Therefore, when a thalamic reticulum infarction occurs, a large quantity of GABA is released due to the sudden death of neurons.^[30]^ We hypothesize that this explosive release of GABA will inevitably lead to early local nervous system activity disorders in the event of cerebral infarction. GABA neurons, through N-type cholinergic receptors, promote increased sleep in patients.^[3]^ Some studies^[31]^ suggest that increased GABA release can play a neuroprotective role after early-stage of ischemia. We hypothesize that when the excess GABA is utilized as an energy source, the patient’s consciousness returns to normal through the other side compensation. However, this speculation needs further confirmation by pathophysiological studies and monitoring of related clinical studies.

## Acknowledgments

We thank *Medjaden* Inc. for the scientific editing of this manuscript.

## Author contributions

YW contributed to the conception of the study and wrote the manuscript. WK and LM contributed significantly to analysis and manuscript preparation. CY and WZ helped perform the analysis with constructive discussions.

**Conceptualization:** Changyou Yin, Wei Zhao, Yanbin Wang.

**Data curation:** Wei Kong, Lei Ma.

**Formal analysis:** Wei Kong, Lei Ma, Changyou Yin, Wei Zhao.

**Methodology:** Yanbin Wang.

**Writing – original draft:** Yanbin Wang.

**Writing – review & editing:** Wei Zhao, Yanbin Wang.

All authors contributed to the manuscript and approved the submitted version.
